# A 13-year old girl with pancytopenia at the presentation of a *Borrelia hispanica* infection: a case report and review of the literature

**DOI:** 10.1186/s13256-017-1225-3

**Published:** 2017-02-27

**Authors:** Irmin Leen, Peggy Bruynseels, Benoît Kabamba Mukadi, Mark van Oort, Machiel van den Akker

**Affiliations:** 1 Department of Pediatrics, Queen Paola Children’s Hospital, Lindendreef 1, 2020 Antwerp, Belgium; 2 Department of Emergency Medicine, ZNA Middelheim, Antwerp, Belgium; 3 Department of Microbiology, ZNA Middelheim, Antwerp, Belgium; 4 Department of Clinical Microbiology, Cliniques Universitaires UCL St-Luc, Brussels, Belgium; 5 Department of Pediatric Hematology Oncology, UZ Brussel, Brussels, Belgium

**Keywords:** *Borrelia hispanica*, Children, Pancytopenia

## Abstract

**Background:**

It is not uncommon that a child with a febrile illness of unknown etiology is admitted to the hospital. When the complete blood count reveals a pancytopenia, the diagnostic process can be a real challenge.

**Case presentation:**

A 13-year girl of Arab-Berber descent presented with abdominal pain and fever after a holiday in northwestern Morocco. A complete blood count revealed a pancytopenia and blood smear test results revealed spirochetes. *Borrelia hispanica* was identified by sequencing the 16S ribosomal ribonucleic acid gene. Our patient was treated with tetracyclines and during this treatment we saw full clinical and hematological recovery.

**Conclusions:**

*Borrelia hispanica* is a known cause of tick-borne relapsing fever and is transmitted to humans through the bite of soft ticks of the genus *Ornithodoros* (*Alectorobius*). Although the link between tick-borne relapsing fever and thrombocytopenia has been documented, there are only a few case reports of tick-borne relapsing fever presenting with pancytopenia. To the best of our knowledge, there is no previous report of *Borrelia hispanica* presenting with pancytopenia.

## Background

Climate change has increased migrant influx and increasing numbers of intercontinental travelers will cause higher prevalence of relatively unknown parasitic diseases in Western Europe. It is therefore not uncommon that a child with a febrile illness of unknown etiology is admitted to the hospital. The unfamiliarity of the pathology makes the diagnostic process a real challenge, especially when the presentation is unusual for an uncommon disease, like the one we present here.

## Case presentation

A 13-year old girl of Arab-Berber descent (Morocco) presented to our emergency department because of abdominal cramps and pain in the right and left iliac fossa for 3 days, accompanied by vomiting and high fever. One week before, she had returned from a 2-month visit in northwestern Morocco. During her stay in Morocco, she was residing in the house of a family member. There was no contact with animals, no history of a tick bite, and she did not visit any parks or forests. She swam in the Mediterranean Sea and in a freshwater swimming pool, but she never went swimming in natural freshwater resources. She had not been sick during her stay and no skin rash was seen. Returning to Belgium, the next day she developed lower abdominal pain, diarrhea, vomiting, and high fever. After 4 days, she presented to the emergency department of our hospital.

Her medical history revealed a right-sided Bell’s palsy in the previous year with a magnetic resonance imaging (MRI) scan that showed a neuritis facialis, and a *Borrelia* serological test result that was negative. She had recurrent episodes of herpes labialis.

**Fig. 1:**
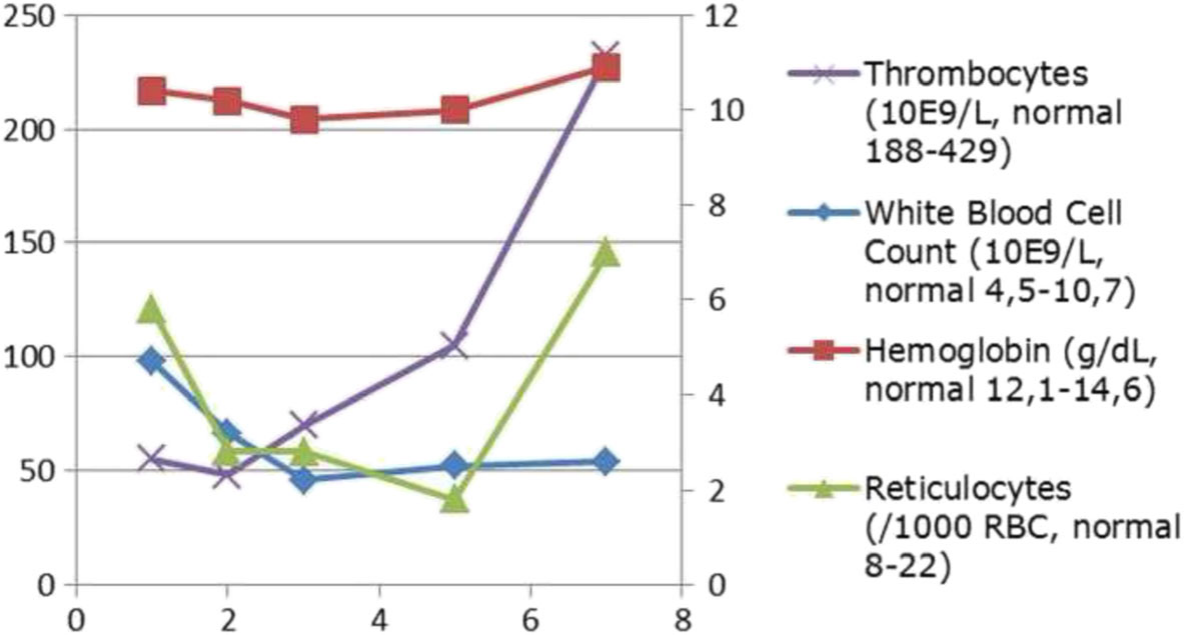
Blood count at diagnosis and response to therapy. *Left y-axis*: thrombocytes. *Right y-axis*: white blood cell count, hemoglobin and reticulocytes. *X-axis*: time in days

**Fig. 2:**
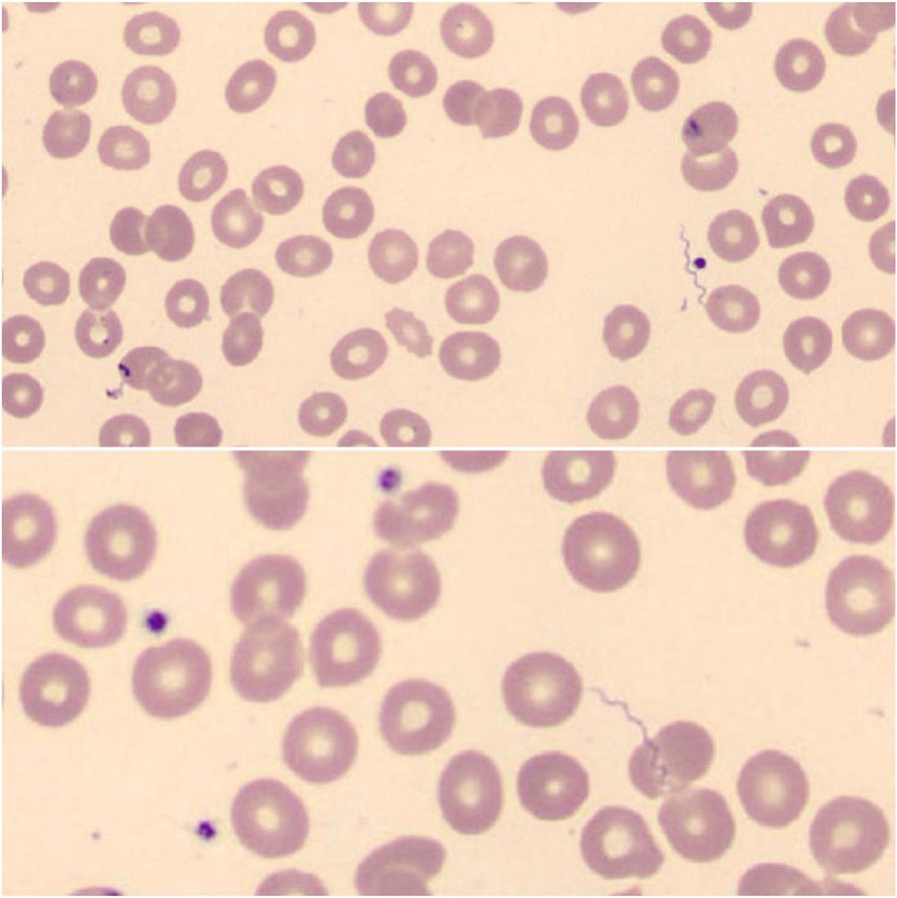
Blood smear test result revealing spirochetes with irregular, wide, open coils suggesting a *Borrelia* infection

A physical examination at presentation showed a mildly sick girl with stable cardiovascular and respiratory parameters, without fever. She had a herpes labialis lesion on her lower lip. Her heart and lung auscultation were normal. An abdominal investigation showed normal bowel sounds and diffuse pain by palpation, most pronounced in the right and left fossa. The initial complete blood count showed a mild, Coombs-negative, normocytic anemia with a hemoglobin of 10.4 g/dL (normal 12.1–14.6 g/dL), reticulocytes 5.8/1000 red blood cells (RBC) (normal 8–22/1000 RBC), leukocytes of 4.7 × 10E9/L (normal 4.5–10.7 × 10E9/L) with a mild neutropenia and mild monocytosis, and thrombocytes of 55 × 10E9/L (normal 188–429 × 10E9/L), with C-reactive protein of 210 mg/L (normal ≤ 5 mg/L). There were no signs of hemolysis with normal lactate dehydrogenase and bilirubin. An abdominal ultrasound scan was normal, but without visualization of the appendix. A urine investigation and thoracic X-ray showed no signs of infection. Since appendicitis could not be ruled out, an appendectomy was done immediately; however, the appendix was not inflamed on visualization. The next day, the complete blood count revealed a more pronounced pancytopenia (see Fig. 1) and the blood smear test result showed some spirochetes. Therefore, the differential diagnosis was narrowed to leptospirosis and borreliosis (see Fig. 2), making the latter more likely given the microscopic and morphological characteristics of the spirochetes. Serological test results for leptospirosis immunoglobulin M and G (IgM and IgG) and *Borrelia burgdorferi* were negative, as well as for ehrlichiosis, anaplasmosis, babesiosis, Rocky Mountain spotted fever and other parasitic infections. The diagnosis of tick-borne relapsing fever was suspected and treatment with tetracycline (intravenous, 20–40 mg/kg/day in four doses) was initiated. Blood and urine cultures did not reveal growth. Sequencing of 1500 bp of the 16S ribosomal ribonucleic acid (rRNA) gene, of which 872 bp was analyzable [on a Genetic Analyzer ABI 3730XL (Applied Biosystems, Invitrogen Life Technologies, Carlsbad, CA, USA), with the BigDye Terminator kit (Applied Biosystems) using a home-brew method], confirmed our diagnosis. Basic Local Alignment Search Tool (BLAST) analysis of the consensus sequence revealed 100% identity with the first 13 propositions representing *Borrelia hispanica* 16s ribosomal RNA gene sequences. The closest match to the next *Borrelia* species was observed with several *Borrelia crocidura* strains showing 99% identity. The nucleotide sequence was submitted to GenBank and obtained the accession KY285287. Our patient was treated with a combination of ceftriaxone (2 g/day in 1 dose, for 3 days) and doxycycline (2 mg/kg/day in 1 dose, for 14 days) during which we saw a full clinical and hematological recovery.

## Discussion

Spirochetes are Gram-negative bacteria with a double membrane and a helicoidal structure. Flagella are present, allowing the spirochete to rotate as it moves. They are divided into three families (Leptospiraceae, Brachyspiraceae and Spirochaetaceae) and are responsible for several diseases in humans, for example leptospirosis, Lyme disease, relapsing fever, syphilis and intestinal spirochaetosis. Tick-borne relapsing fever (TBRF) is an infection caused by spirochetes of the genus *Borrelia*, transmitted to humans through the bite of soft ticks (*Ornithodoros* species). It is caused by at least 16 distinct *Borrelia* species throughout the world [1, 2]. *Borrelia* species are Gram-negative helical bacteria that normally measure 0.2 to 0.5 μm in width and 5 to 20 μm in length. They are very hard to culture, but visible with dark-field and light microscopy. They have the corkscrew shape typical of all spirochetes [3]. Each *Borrelia* species associated with relapsing fever appear to be specific to its tick vector [2].

First signs of TBRF are usually observed between 4 and 14 days after the tick bite, with an acute onset of high fever, headache, arthralgia, myalgia, neck stiffness, and abdominal complaints [4]. Case fatality rate is 2–5% without treatment. Severity depends on *Borrelia* species*,* inoculum density, and underlying medical condition. Children and women appear to have a more intense course of disease [5]. The primary episode usually lasts 3 days and is followed, after a fever-free interval of 7 days, by multiple other alternating episodes, often shorter and milder. During the febrile periods, numerous *Borreliae* are circulating in the blood and diagnosis can be made by observation of spirochetes on thin- or thick-blood smears with dark-field microscopy or with conventional microscopy after Giemsa, Wright or Diff-Quick staining [5, 6].

*Borrelia* cultures have not been widely used due to the low sensitivity range. Molecular methods are used with increasing frequency and offer the possibility of genus and even species identification [7]. As such, *Borrelia hispanica* has been detected and isolated from specimens obtained in Northern Africa and Southern Europe, including Morocco, Spain, Portugal, Greece and Cyprus and is held responsible for 20.5% of patients with unexplained fever in northwestern Morocco [1, 8]. The disease caused by *Borrelia hispanica* is one of the less severe TBRFs, with neurological signs in less than 5% of cases [9]. The preferred treatment for TBRF is tetracycline or doxycycline. When contraindicated, erythromycin is the alternative. In very sick patients (with neurologic symptoms) intravenous ceftriaxone can be added. No exact data is available in the literature about the association of TBRF and thrombocytopenia at presentation, but it is not uncommon [4]. In contrast, TBRF presenting with pancytopenia is rare and only reported in a few case reports. Badger *et al*. described an infection with the recently discovered *Borrelia miyamotoi* and pancytopenia, and Chowdri *et al.* presented a case of a 59-year-old woman with borreliosis and pancytopenia. Her bone marrow was packed with *Borrelia hermsii* [3, 10]. Pancytopenia is a common manifestation of many tick-borne diseases; however the pathogenesis is poorly understood, possibly resulting from decreased bone marrow production, consumption due to widespread endothelial damage or due to immune-mediated destruction. Data on the prevalence of thrombocytopenia, neutropenia or anemia with *Borrelia hispanica* TBRF is not available and to the best of our knowledge, no reports of *Borrelia hispanica* presenting with pancytopenia have been published. *Borrelia hispanica* is transmitted through soft ticks, making co-infection of other tick-borne bacterial diseases like anaplasmosis or babesiosis, which are known to present with cytopenias, but transmitted through hard ticks of the *Ixodes* species, unlikely.

## Conclusions

We report a case of a patient with *Borrelia hispanica* tick-borne relapsing fever presenting with abdominal pain, fever and pancytopenia. TBRF is a rare disease in Europe. It is found in some Mediterranean countries, and is a frequent cause of fever in northwestern Morocco. Therefore it should be in the differential diagnosis of travelers returning from these areas presenting with unexplained fever. Although the link between TBRF and thrombocytopenia has been documented, there are only a few cases of TBRF presenting with pancytopenia. To the best of our knowledge, this is the first report of *Borrelia hispanica* presenting with pancytopenia.
